# Supplementary material for: Solid matrix-assisted printing for three-dimensional structuring of a viscoelastic medium surface
Source: Nat Commun. 2019 Oct 11;10:4650. doi: 10.1038/s41467-019-12585-9 (PMC6789121; doi:10.1038/s41467-019-12585-9)
Supplement: Supplementary file 1 — Supplementary Information [file 41467_2019_12585_MOESM1_ESM.pdf]

Supplementary Information

Solid Matrix-Assisted Printing for Three-Dimensional Structuring of a Viscoelastic Medium Surface

Shin et al.

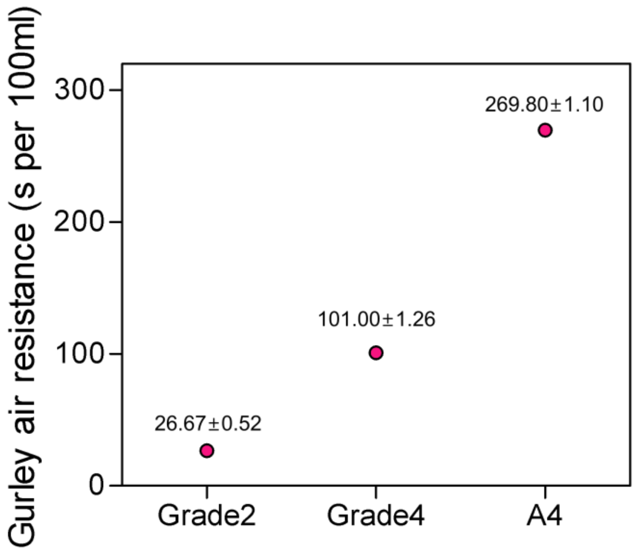

Supplementary Figure 1. Gurley air permeability of paper materials (filter paper grades 2, 4, and A4). Data are presented as mean ± s.d. (n=5).

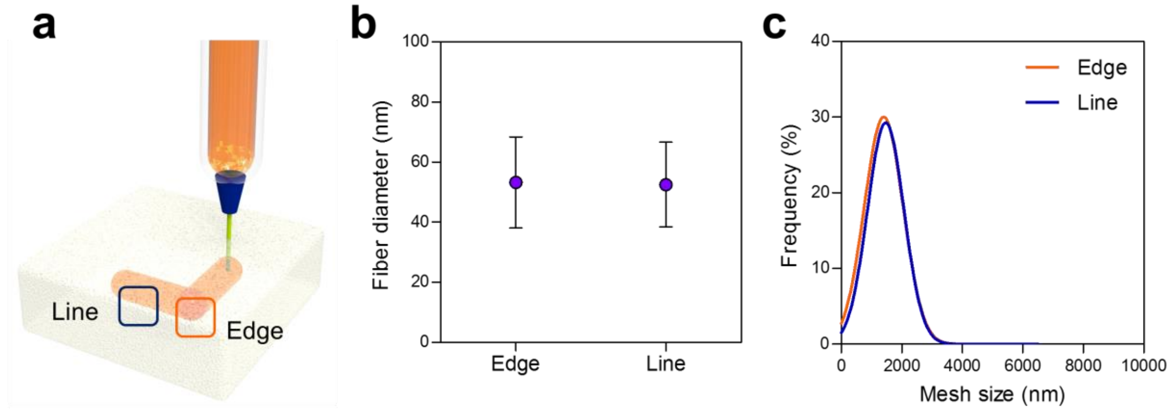

Supplementary Figure 2. BC network properties depending on the printed structure. a Schematic illustration of angled line printing. b Fiber diameter of BC at edge part and line part. c Mesh size of BC at edge part and line part. Data are presented as mean ± s.d. (n=3 in b).

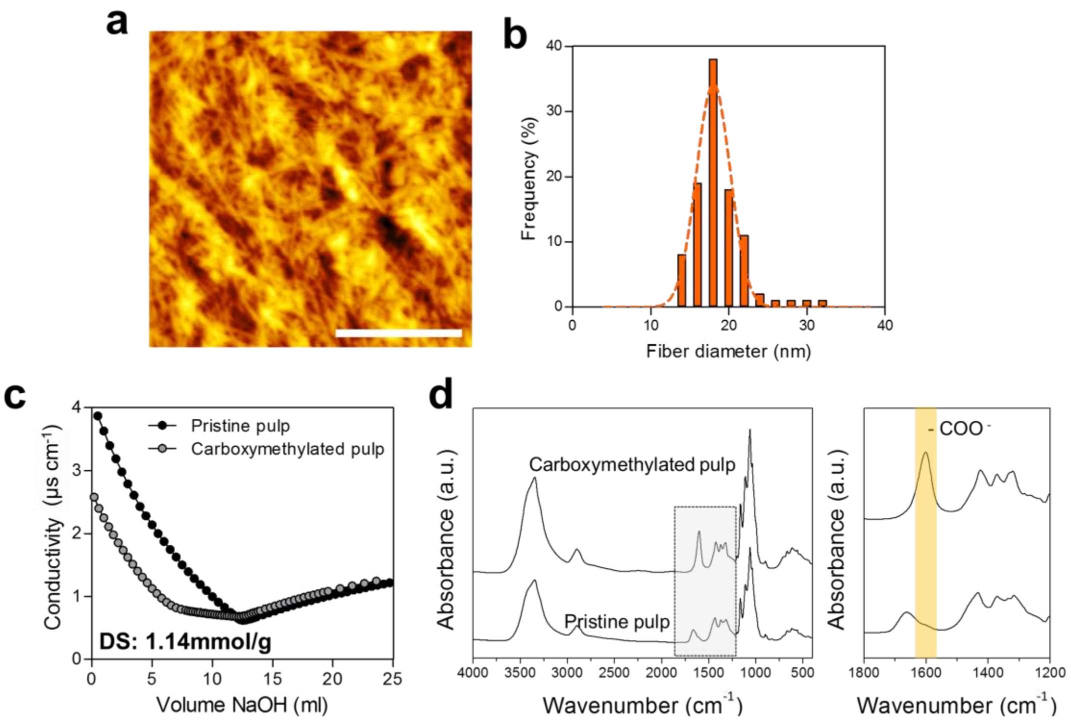

Supplementary Figure 3. Characterization of CM-CNF. a AFM image of nanofibrillated CM-CNF. b Fiber diameter distribution of CM-CNF. The average fiber diameter was 18.6 nm. c Conductivity titration curve of pristine pulp cellulose, and the carboxylated pulp cellulose. d FTIR spectra of pulp cellulose before and after carboxymethylation. The degree of substitution was 1.14 mmol g^{-1} and the yield of CM-pulp was 80%. The viscosity average molecular weight of CM-CNF was 70048. Scale bar is 500 nm in a.

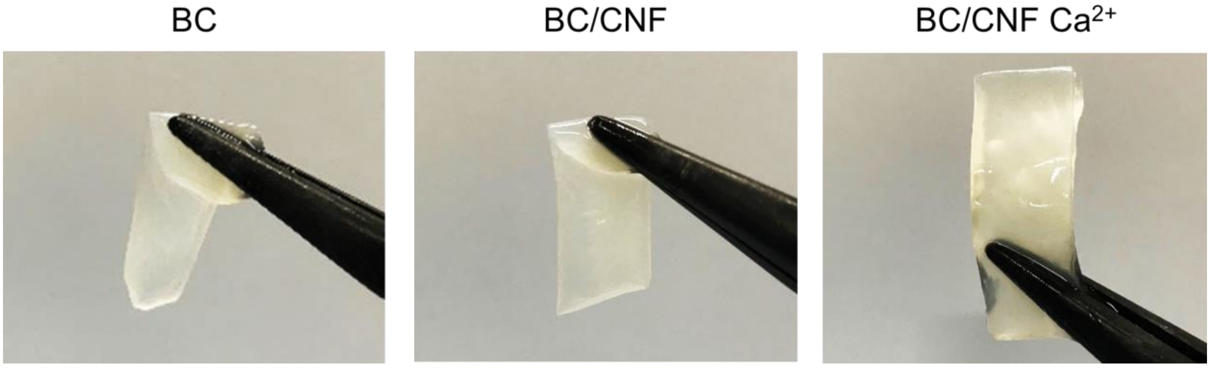

Supplementary Figure 4. Dimensional stability of pristine BC, BC/CNF hydrogel, and BC/CNF hydrogels treated with 1% calcium ion solution.

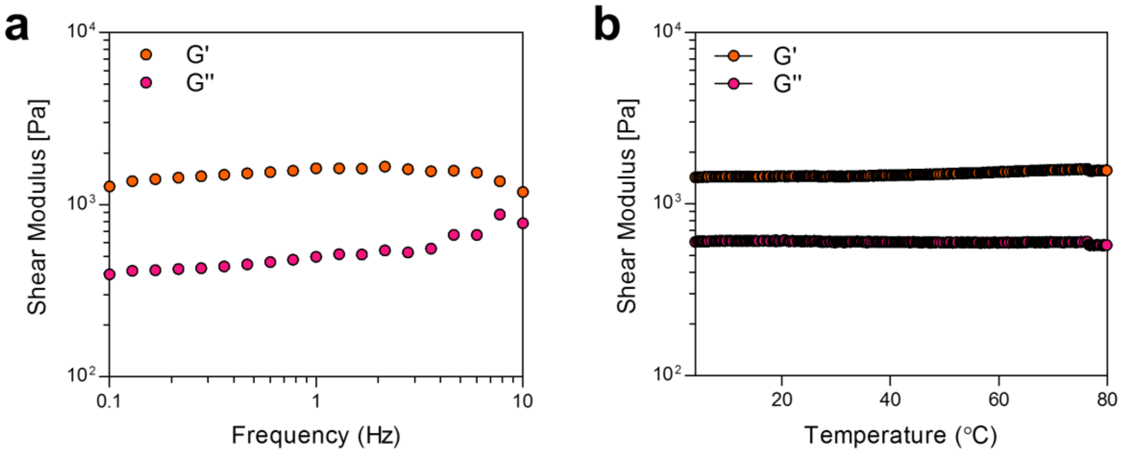

Supplementary Figure 5. Rheological properties of CNF/BC treated with Ca^{2+}. a Frequency sweep test of CNF/BC hydrogel. b Temperature sweep test of CNF/BC hydrogel.
